# Hyaluronic Acid-Decorated Laponite^®^ Nanocomposites for Targeted Anticancer Drug Delivery

**DOI:** 10.3390/polym11010137

**Published:** 2019-01-14

**Authors:** Tingting Jiang, Guangxiang Chen, Xiangyang Shi, Rui Guo

**Affiliations:** Key Laboratory of Science & Technology of Eco-Textile, Ministry of Education, College of Chemistry, Chemical Engineering and Biotechnology, Donghua University, Shanghai 201620, China; tingting_jiang1993@foxmail.com (T.J.); gxchen0429@163.com (G.C.)

**Keywords:** LAP, hyaluronic acid, doxorubicin, CD44 receptor targeted

## Abstract

In this study, hyaluronic acid (HA), a natural polysaccharide that can specifically bind to CD44 receptors, was conjugated onto laponite^®^ (LAP) nanodisks for the encapsulation and specific delivery of the anti-cancer drug doxorubicin (DOX) to CD44-overexpressed cancer cells. The prepared LM-HA could encapsulate DOX efficiently and release drug in a continuous manner with pH-responsiveness. In vitro cell viability assay proved that LM-HA had good biocompatibility, and drug-loaded LM-HA/DOX exhibited targeted anti-tumor effects against HeLa cells with CD44 receptors overexpressed. In addition, the flow cytometric detection and confocal laser scanning microscope results confirmed that LM-HA/DOX could be specifically internalized by HeLa cells via CD44-mediated endocytosis. Therefore, the HA-modified LAP nanodisks with high drug loading efficiency, pH-sensitive drug release properties and CD44 targetability might be an efficient nanoplatform for cancer chemotherapy.

## 1. Introduction

Chemotherapy is one of the most widely used and indispensable tools in cancer treatments. Small molecular anticancer drugs could interfere with cell mitosis and effectively inhibit cell proliferation, but the lack of specific selectivity to tumor cells and fast body metabolism may cause severe side effects and low drug bioavailability, limiting their clinical applications [1]. To address these problems, nanosized drug delivery systems have been developed to prolong the circulation time in the blood, and enhance the drug accumulation in the tumor via the enhanced permeability and retention (EPR) effect [2]. In addition, a series of tumor-targeting moieties were functionalized on the surface of nanocarriers to improve the specific delivery and uptake of drugs by cancer cells, including RGD (Arg-Gly-Asp) [3], folic acid (FA) [4], phenylboronic acid (PBA) [5], hyaluronic acid (HA) [6], etc. [7,8,9]. However, it remains a challenge to construct a desired vehicle with a high drug loading efficiency, a controlled drug release profile, and specific targeting to cancer cells [10].

Until now, various kinds of inorganic and organic materials have been used in constructing nano-drug delivery systems, such as liposomes [11], dendrimers [12], polymer micelles [13], nanogels [14], mesoporous silica nanoparticles (MSN) [15], carbon nanotubes (CNTS) [16] and nanoclays [17]. Among them, laponite (LAP) nanodisks were considered as one of the most promising delivery vehicles due to their unique structure, good stability, excellent biocompatibility and ease of surface functionalization [18]. As a type of artificial nanoclay, LAP has a similar composition and layered structure to native hectorite, and can be degraded into nontoxic products under physiological conditions [19]. Moreover, LAP can be dispersed in aqueous solution as nanodisks with about 25 nm in diameter and 1 nm in thickness, which means that LAP has a highly-specific surface area and strong cationic exchange capability for the effective encapsulation of various drug molecules [20], such as doxorubicin (DOX) [21], amoxicillin [22], tetracycline [23], and dexamethasone [24]. In our previous work, LAP has been applied to encapsulate the positively-charged anticancer drug DOX with pH-sensitive drug-release properties and an effective drug encapsulation efficiency of 98%. The prepared LAP/DOX complexes showed a more efficient therapeutic efficacy compared to free DOX in vitro, by the virtue of their nano-size [20]. Moreover, tumor targeting agents could be further decorated on LAP to improve specific targeting to cancer cells. For example, DOX-loaded, FA-modified LAP nanocomposites displayed significantly enhanced therapeutic efficacy in treating cancer cells overexpressing FA receptors [25]. PEG-lactobionic acid modified LAP possessed great colloidal stability and exhibited selective cytotoxic properties to targeted hepatocarcinoma cells overexpressing asialoglycoprotein receptors [26].

A cluster of differentiation-44 (CD44) is a transmembrane glycoprotein over-expressed in a variety of solid tumors, such as breast cancer, gastric carcinoma, hepatocarcinoma, and melanoma [27]. Hence, many studies have focused on targeting the overexpression of CD44 receptors to improve the specific deliver and endocytotic uptake of drugs in cancer cells [28,29]. Hyaluronic acid is recognized as the primary CD44 binding molecule because of the existence of the binding site, which connects HA and CD44 receptors on the surface of cancer cells [30]. For instance, Zhang et al. synthesized HA-modified, single-walled carbon nanotubes to load DOX. In vitro experiments demonstrated that the drug delivery system could induce significantly higher cytotoxicity against a human cervical cancer cell line (HeLa cells) overexpressing CD44 receptors than normal fibroblasts, and histological examinations proved their lower toxicity to vital organs in comparison with DOX [31]. He et al. used HA to functionalize PS/CaCO_3_/DNA nanoparticles (HNP) for targeted gene delivery. After 4 h of incubation with HeLa cells, HNP displayed a significant higher mean fluorescence intensity than non-targeted NPs, indicating that the enhanced cell uptake of HNP is caused by CD44-mediated cellular uptake [32]. In Cheng’s work, cisplatin-incorporated Cy5.5-PEG-*g*-HA nanoparticles could selectively inhibit the proliferation of HeLa cells, and exhibited an efficacious accumulation in tumors [33]. Therefore, HA modification could increase cell uptake and preferential accumulation in tumors overexpressing CD44 receptors, resulting in reduced residual toxicity and an improved therapeutic effect [34].

In this study, HA-modified LAP nanocomposites were synthesized to load the antitumor drug DOX for targeted delivery to cancer cells overexpressing CD44 receptors. The structure of the prepared LM-HA was characterized by ^1^H NMR, FT-IR spectrometry, thermal gravimetric analyses (TGA), dynamic light scattering (DLS) and UV-vis spectrometry. The release profile of LM-HA/DOX nanocomposites was investigated under both acidic and physiological conditions. Furthermore, CD44 overexpressed HeLa cells were utilized as model cells to estimate the specific targeting and antitumor efficacy of LM-HA/DOX nanocomposites by 3-(4,5-Dimethylthiazol-2-yl)-2,5-diphenyltetrazolium bromide (MTT) assay, flow cytometric analysis, cell morphology and confocal laser scanning microscopic (CLSM) observation.

## 2. Experiment 

### 2.1. Preparation and Characterization of DOX-Loaded Nanocomposites

According to the previous literature [35], 50 mg of laponite^®^ (LAP) powder was dissolved in 5 mL of ultrapure water and magnetically stirred at 50 °C overnight to obtain completely LAP aqueous solution. Then 1 mL APMES aqueous solution (20 mg/mL) was slowly injected into the LAP aqueous solution and stirred at 50 °C for 16 h. The resulting solution was then dialyzed against a dialysis bag with a molecular weight cutoff of 8000–14,000 for 3 days (2 L, 3 times per day). After dialysis was completed, the product in the dialysis bag was transferred to a 50 mL EP tube and stored at 4 °C to obtain LM-NH_2_.

Then, HA (84.35 mg) was dissolved in ultrapure water (5 mL) and activated by EDC (104.1 mg) and NHS (62.51 mg) at room temperature for 3 h. Next, the activated HA aqueous solution was added to LM-NH_2_ (6.276 mL, 52.33 mg) and stirred for 3 days. Finally, the reaction mixture was extensively dialyzed against water (2 L, 3 times per day) for 3 days using a dialysis bag with a molecular weight cutoff of 8000–14,000 to obtain targeted LM-HA. 

The DOX/HCl powder was dissolved in ultrapure water to obtain DOX aqueous solution (2 mg/mL). Then, LM-HA aqueous solution (6 mg/mL) was mixed with DOX aqueous solution in a mass ratio of 3:1. The drug loaded LM-HA/DOX can be obtained by magnetically stirring for 24 h in darkness. Subsequently, the solution was purified by centrifugation (15 min, 8500 rpm) and washed 3 times with ultrapure water to achieve drug-loaded LM-HA/DOX nanocomposites. The drug loading efficiency and loading capacity can be calculated using Equations (1) and (2), respectively:

Loading efficiency = (M_t_/M_0_) × 100% (1)

Loading capacity = M_t_/(M_t_ + M_L_) × 100% (2)

where M_t_, M_0_ and M_L_ stand for the mass of the encapsulated DOX, the initial DOX, and the LM-HA/DOX nanocomplexes, respectively.

Details of these methods can be found in the Supplementary Materials, including materials and characterization techniques, and in vitro drug release.

### 2.2. Cell Culture

HeLa cells were continuously cultured in a 75 cm^2^ tissue culture flask with 15 mL Dulbecco’s modified Eagle’s medium (DMEM) containing 10% FBS, 100 U/mL penicillin, and 100 μg/mL streptomycin in a humidified incubator with 5% CO_2_ at 37 °C. HA pre-treated HeLa cells were obtained by pre-treating HeLa cells with HA-containing DMEM medium (3 mM) for 1 h before the addition of LM-HA or LM-HA/DOX [36,37].

### 2.3. In Vitro Cytotoxicity Assay and Cell Morphology Observation

HeLa cells were collected and seeded in the 96-well plate at the density of 1 × 10^4^ cells/well, with 100 μL DMEM medium for 24 h. The next day, the medium was discarded and replaced with 100 μL of fresh medium, containing 10 μL phosphate buffered saline (PBS) of LM-HA nanodisks at different final concentrations (0, 1.5, 3.0, 6.0, 12, 24, 48 and 96 μg/mL, respectively) (n = 5). PBS was used as control. After a 24 h culture to bring the cells to confluence, the medium was discarded and cells were rinsed with PBS 3 times, followed by the addition of 90 μL medium and 10 μL MTT solution, and continued to incubate for 4 h. Then the original medium was washed out and 100 μL DMSO solution was added to each hole in shaking table for 20 min. A Thermo Scientific Multiskan MK3 ELISA reader (Waltham, MA) was used to record the absorbance of each well at 570 nm. 

To check the therapeutic efficacy of LM-HA/DOX nanocomposites, HeLa cells and HA pre-treated HeLa cells were incubated with DOX and LM-HA/DOX at different DOX concentrations (0.5, 1.0, 2.0, 4.0 and 8.0 μg/mL) (n = 5) for 24 h. The morphology of cells was observed by using a Leica DM IL LED inverted phase contrast microscope (Wetzlar, Germany) with a magnification of 100× for each sample. MTT assay was also applied to evaluate the cell viabilities against a standard protocol.

### 2.4. Cellular Uptake 

Cover slips with a diameter of 14 mm were pretreated with 5% HCl, 30% HNO_3_, and 75% alcohol, placed in 24-well tissue culture plates, and then soaked by medium for 24 h. HeLa cells were seeded into each well with a density of 1 × 10^5^ cells/well. After culturing for 24 h to bring the cells to attach onto the cover slips, the cells were treated with 0.3 mL fresh medium, which contained free DOX and LM-HA/DOX at a final DOX concentration of 0.5 μg/mL, and then were cultured for another 4 h. After that, the medium was discarded, and the cells were rinsed with PBS 3 times. Then the cells were fixed with glutaraldehyde (2.5%) for 15 min at 4 °C and counterstained with DAPI (1 μg/mL) for 15 min at 37 °C, using a standard procedure. Finally, the mean fluorescence intensity was observed by a confocal laser scanning microscope using a 63 × oil-immersion objective lens (CLSM, Carl Zeiss LSM 700, Jena, Germany). 

The targeting specificity of LM-HA/DOX nanocomposites was quantitatively analyzed via flow cytometry. The HeLa cells were collected and seeded in the 6-well plate at the density of 4 × 10^5^ cells/well for 24 h. Then, the medium was replaced with 1 mL fresh medium containing free DOX and LM-HA/DOX at a final DOX concentration of 0.5 μg/mL, then cultured for a further 4 h. After that, the medium was discarded and the cells were rinsed with PBS 3 times, trypsinated, centrifuged, and resuspended in 1 mL PBS. The intensity of DOX fluorescence was measured using a Becton Dickinson FACScan flow cytometer (FCM, BD Biosciences, Franklin Lake, NJ, USA).

## 3. Results and Discussion

### 3.1. Synthesis and Characterization of LM-HA/DOX

**Scheme 1:**
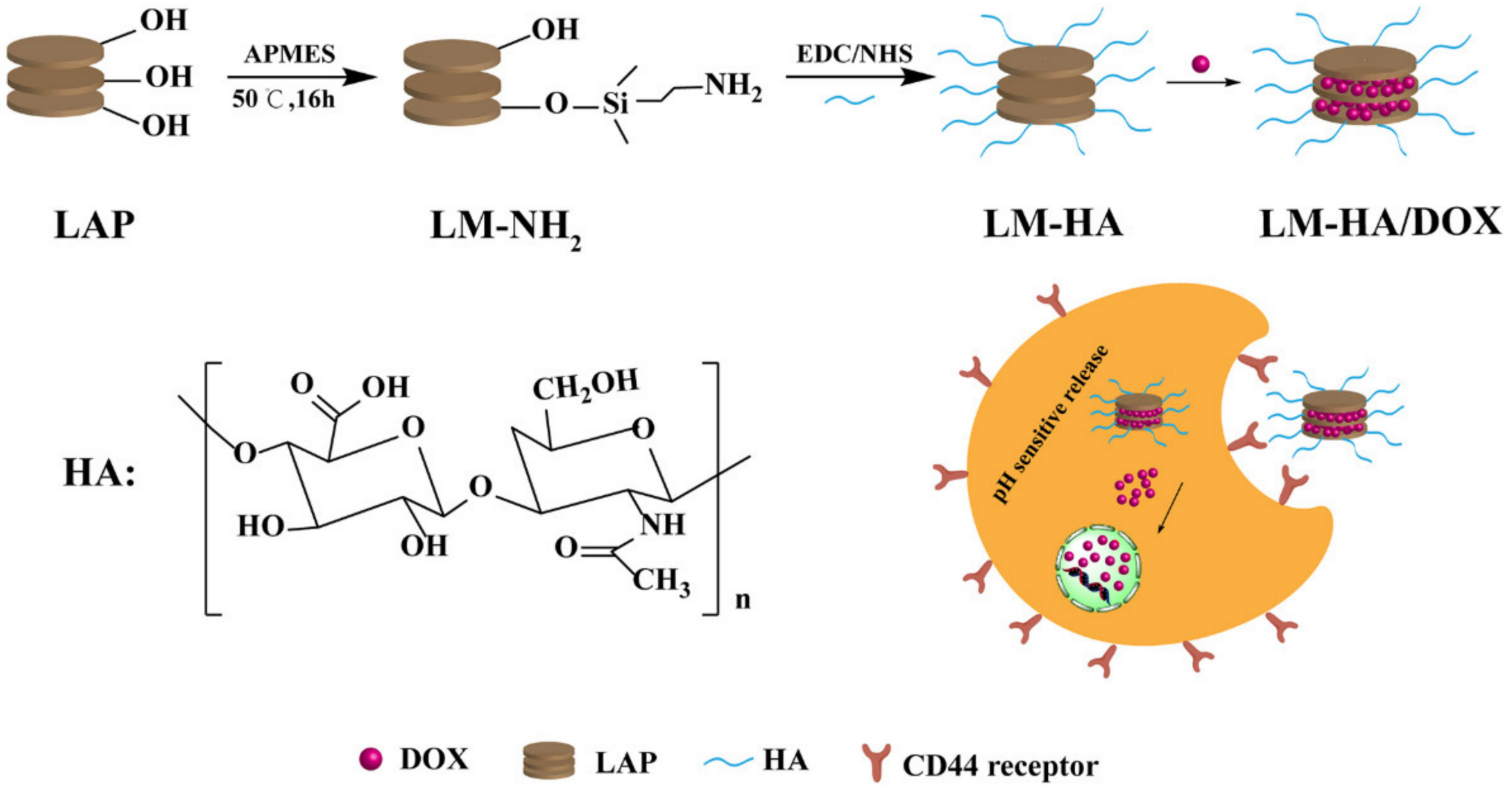
Schematic illustration of the preparation of LM-HA/DOX.

In this study, LAP was first silanized using 3-aminopropyldimethylethoxysilane (APMES) to introduce amine groups on the surface, and then hyaluronic acid was conjugated by the reaction between their carboxyl groups and amine groups on LM via 1-ethyl-3-(3-dimethylaminopropyl) carbodiimide hydrochloride (EDC) chemistry. Finally, the formed LM-HA nanodisks were utilized to load the chemotherapeutic agent doxorubicin by physical absorption (Scheme 1). 

**Figure 1:**
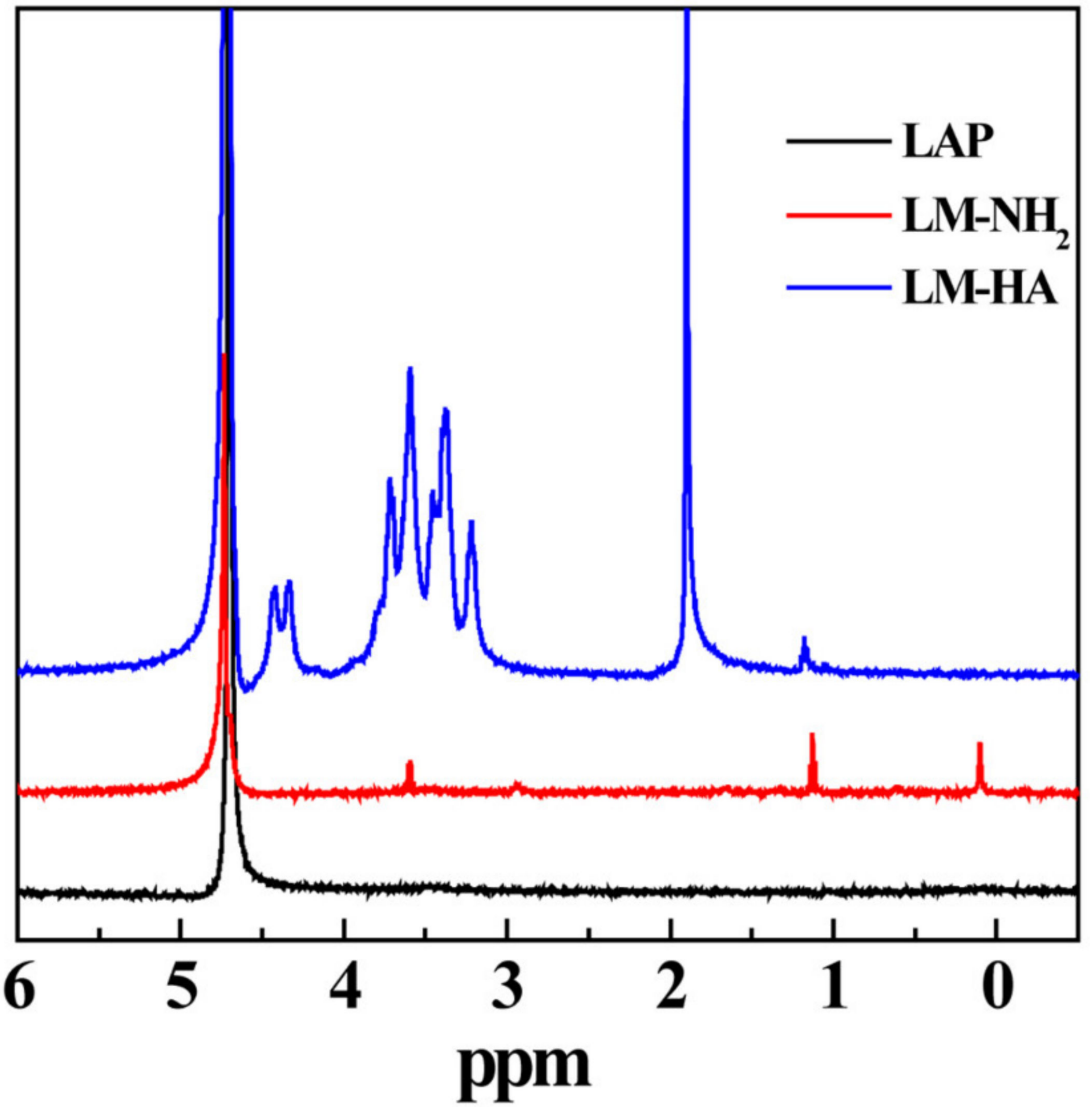
^1^H NMR spectra of laponite nanodisks (LAP), LM-NH_2_ and LM-HA.

The structure of LM-NH_2_ and LM-HA were evaluated by ^1^H NMR, as shown in Figure 1. Compared to pristine LAP, the emergence of peaks at 1.2, 2.8, and 3.6 ppm in the spectrum of LM-NH_2_ were associated to the silane coupling agent APMES, indicating the successful conjugation of APMES on LAP [38]. After the modification of HA, LM-HA displayed a prominent peak at 1.9 ppm in the spectrum, which was attributed to -CH_3_ protons of HA moieties [39]. This result demonstrated that HA was successfully conjugated on LAP via EDC reaction between the carboxyl group of HA and the amine group on LM-NH_2_.

**Figure 2:**
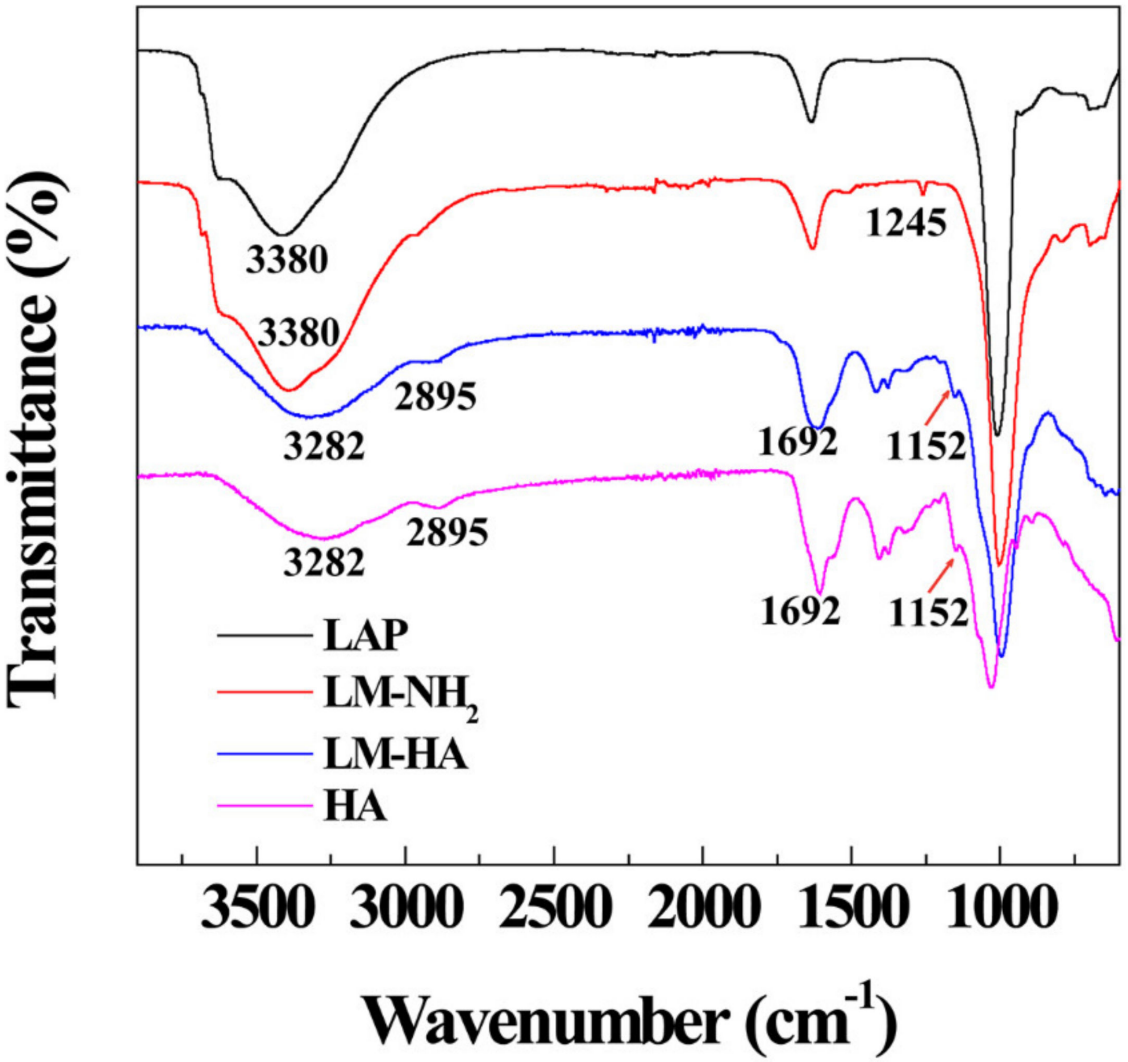
FT-IR spectra of LAP, LM-NH_2_, LM-HA, and HA.

The successful synthesis of LM-HA was also verified by FT-IR, as seen in Figure 2. Compared with LAP, LM-NH_2_ displayed a typical peak at 1245 cm^−1^ triggered by the asymmetric stretching vibration of the Si-O-Si bond, indicating that APMES were successfully decorated onto LAP [21]. After the modification of HA, there were three kinds of prominent peaks at 1692 cm^−1^, 2895 cm^−1^ and 1152 cm^−1^. They were assigned to the C=O stretching vibration of the carboxyl group and the amide I band, the symmetric stretching vibration of C-H, and the C-O stretching of the proteoglycan sugar ring of HA, respectively [40]. Hence, the FT-IR result confirmed the successful conjugation of the targeting agent HA on LAP. 

**Figure 3:**
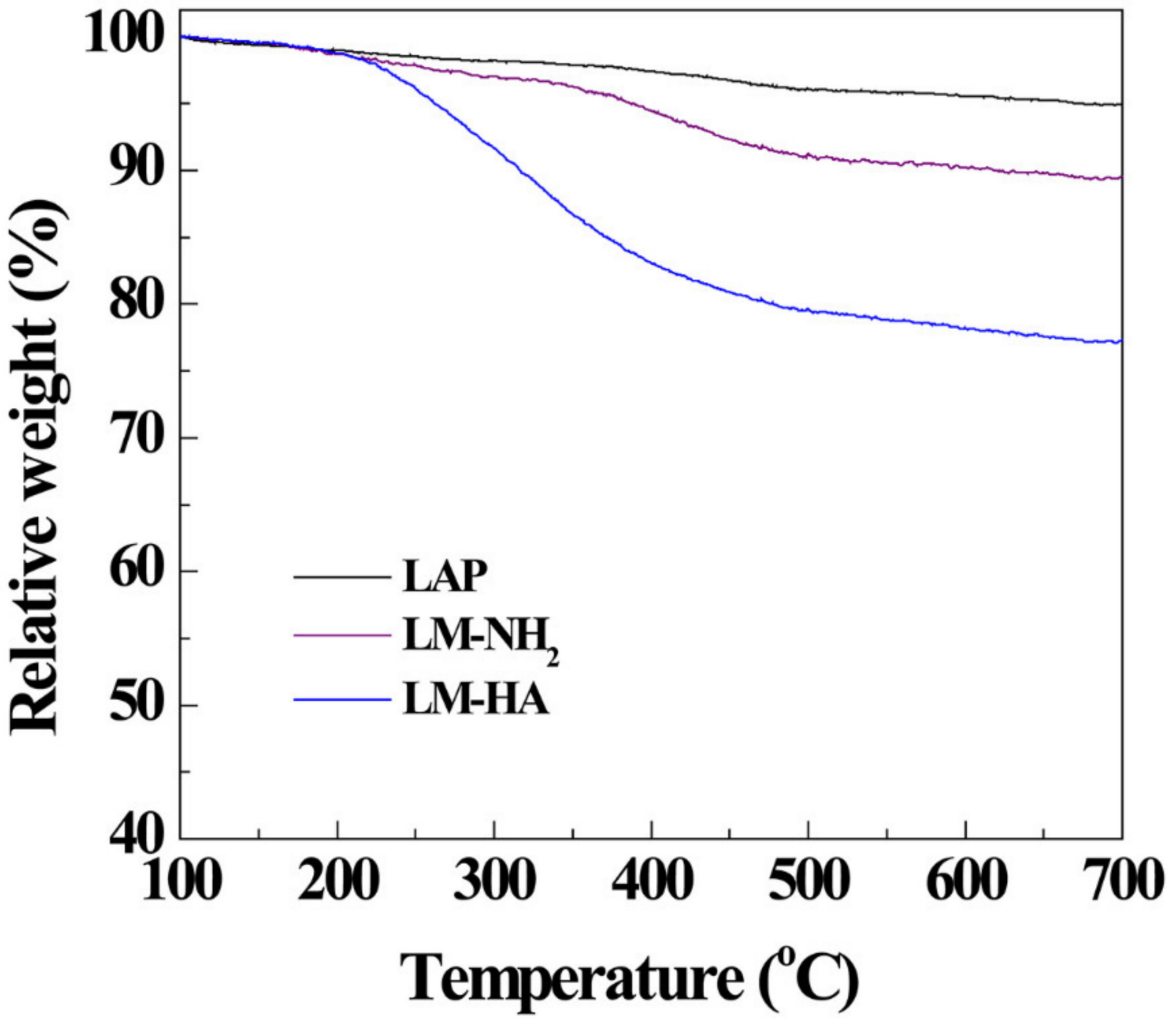
TGA curves of LAP, LM-NH_2_, and LM-HA, respectively.

TGA was then used to quantify the component of LM-HA by measuring the weight loss of LAP, LM-NH_2_, and LM-HA from 100 °C to 700 °C (Figure 3). Compared with the pristine LAP, LM-NH_2_ showed a slight weight loss of 5.79% during the heating process, which was attributed to the thermal decomposition of the APMES [21]. This result demonstrated the conjugation of APMES on LAP. LM-HA exhibited a higher weight loss of 22.70%, and by deducting the residue weight of APMES, the amount of HA modified on LAP was about 13.80% of the final LM-HA product. Therefore, the TGA result further verified the successful synthesis of LM-HA. 

Furthermore, zeta potential measurement and DLS were used to evaluate the change of hydrodynamic and surface potential after modification (Table 1). After silanization, the hydrodynamic diameter increased from 86.5 nm of pristine LAP to 184.0 nm of LM-NH_2_, and the zeta potential surged from −39.6 mV to −12.0 mV, due to the introduction of amine groups on the surface. After the modification of HA, LM-HA showed a lower surface potential of −14.1 mV, which was attributed to the inherent negative charge of HA moieties on surface. Meanwhile, the hydrodynamic size of LM-HA expanded to 282.6 nm because of the conjugation of hydrophilic HA chains, which may provide additional colloidal stability for nanocomposites. 

### 3.2. DOX Encapsulation and Release

**Figure 4:**
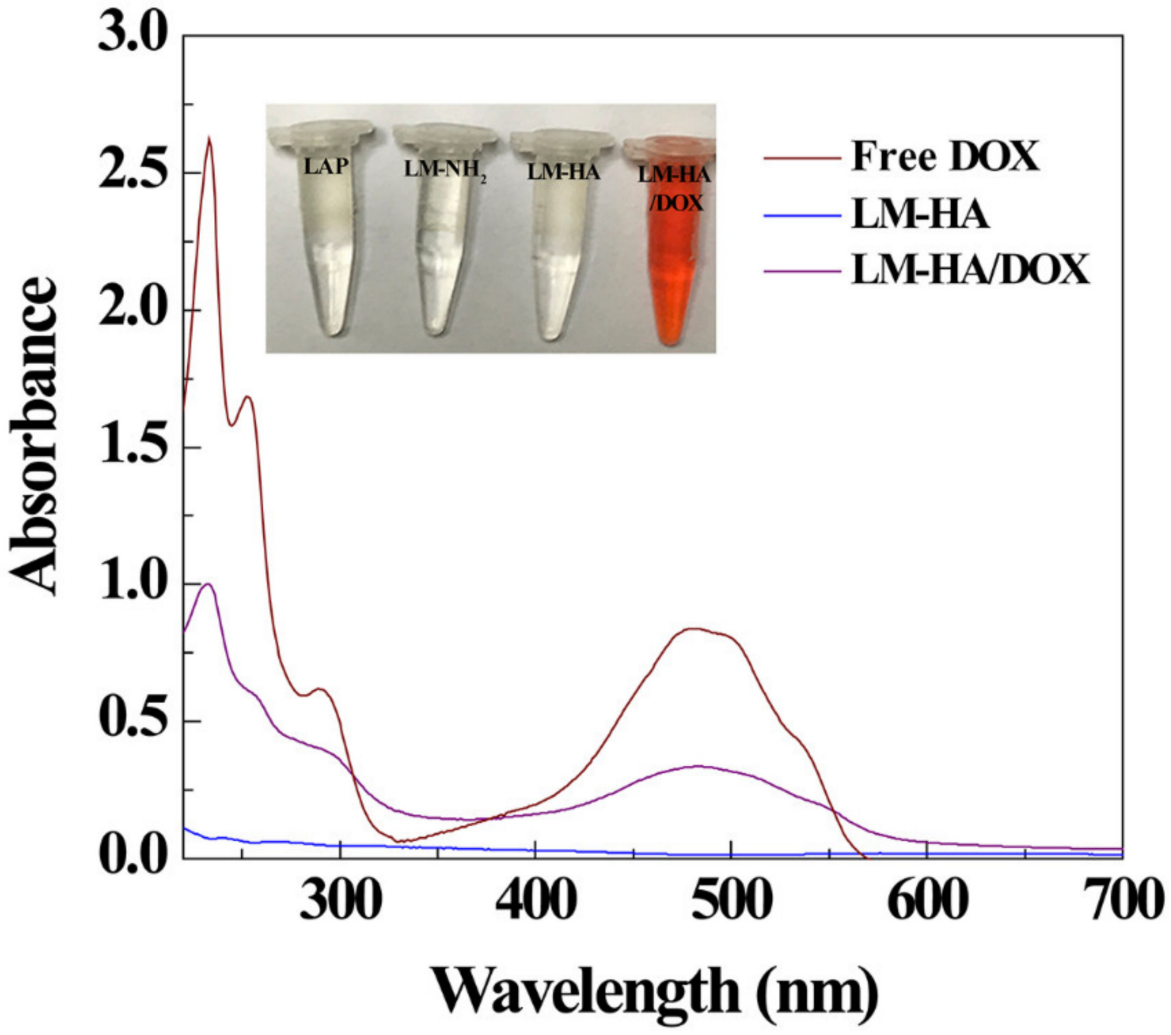
UV-vis spectra of free doxorubicin (DOX), LM-HA, and LM-HA/DOX, respectively.

In this study, LM-HA was used to load anticancer drug DOX by mixing the aqueous solution of LM-HA and DOX for 24 h. UV-vis spectroscopy test was conducted to confirm the encapsulation of DOX into LM-HA nanodisks (Figure 4). Obviously, the solutions of LM-HA did not show any absorbance over 400 nm, while drug-loaded materials displayed an obvious DOX-related absorbance peak at 480 nm, demonstrating that LM-HA could encapsulate DOX effectively [41]. Although the size of LM-HA/DOX expanded to 412.7 nm after drug loading, they still exhibited good stability in PBS, culture medium (DMEM) and fatal bovine serum (FBS) for two weeks (Table S1 and Figure S1). The drug loading efficiency and loading capacity was calculated to be 85.1 ± 1.5% and 22.1 ± 0.22% respectively, using a standard calibration curve. The reason for slightly reduced drug loading efficiency compared to our prior study may be that the increase of surface potential after modification caused a decrease of adsorbed drugs onto LAP via electrostatic interactions [20]. 

**Figure 5:**
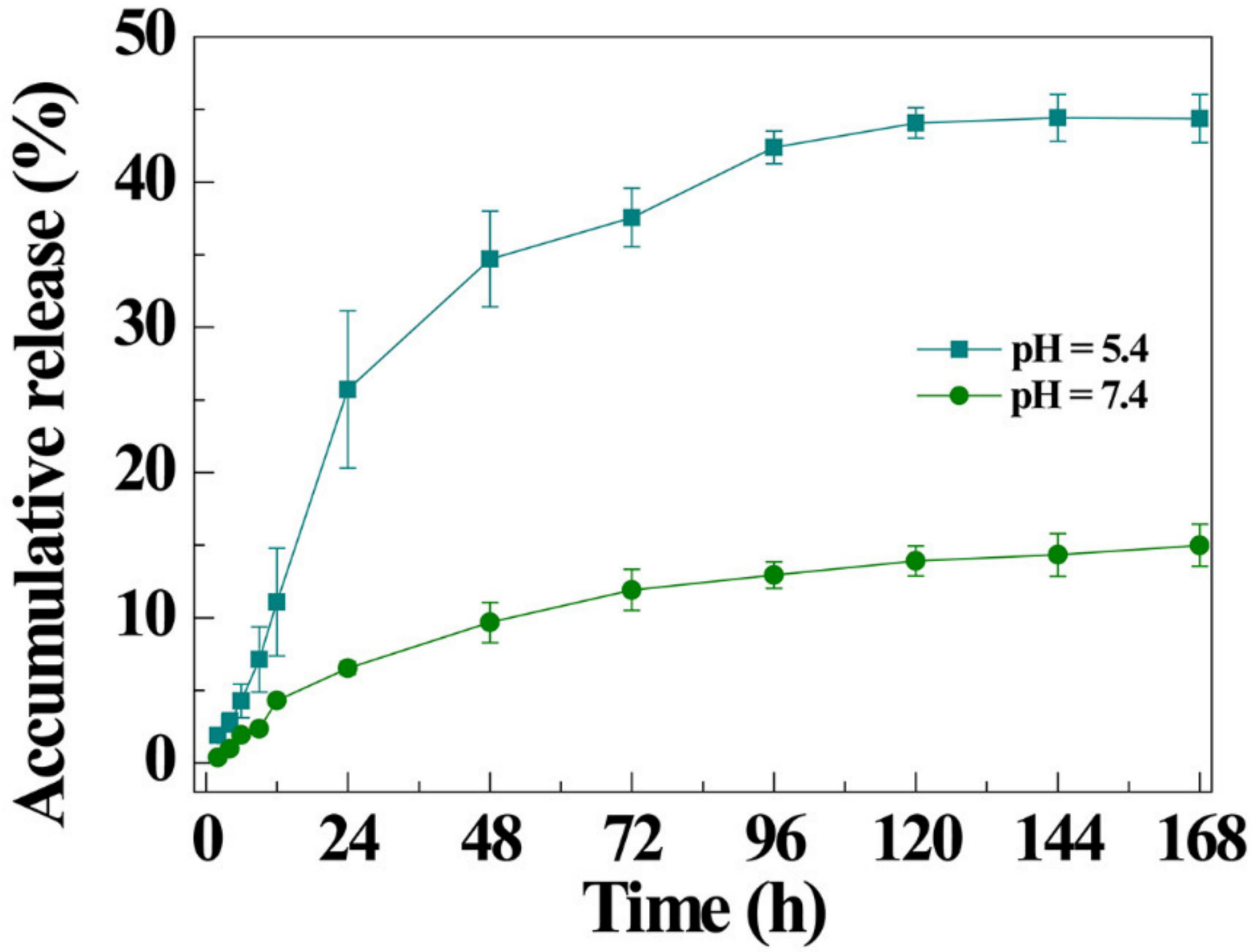
In vitro DOX release profile of LM-HA/DOX at 37 °C under pH 5.4 and 7.4 conditions.

As seen in Figure 5, the release profiles of LM-HA/DOX at pH 5.4 or 7.4 were investigated. Obviously, at a weak acid condition, LM-HA/DOX initially exhibited a fast release of DOX in the first 48 h, and then a sustained release for a long duration. After 168 h, LM-HA/DOX nanocomposites released about 44% of DOX at pH 5.4. The two-phase release profile of LM-HA/DOX may help to restrain the proliferation of cancer cells immediately after administration and keep a relatively high drug concentration for continuous chemotherapy. In contrast, at pH 7.4 condition, the drug release rate was very low, and around 13% of DOX was desorbed after 168 h. The reason for the pH-sensitive release property of LM-HA/DOX may be the good solubility of the salt form (DOX.HCl) of DOX under acidic pH conditions, and its hydrophobic neutral structure under physiological pH conditions [42]. Considering the slightly acidic microenvironment in tumors, a drug released in a sustained manner with pH-responsiveness may be beneficial for chemotherapy, and the decreased leakage of the drug during circulation in physiological conditions may decrease the side effect of DOX. 

### 3.3. Targeted Therapeutic Efficacy of LM-HA/DOX

**Figure 6:**
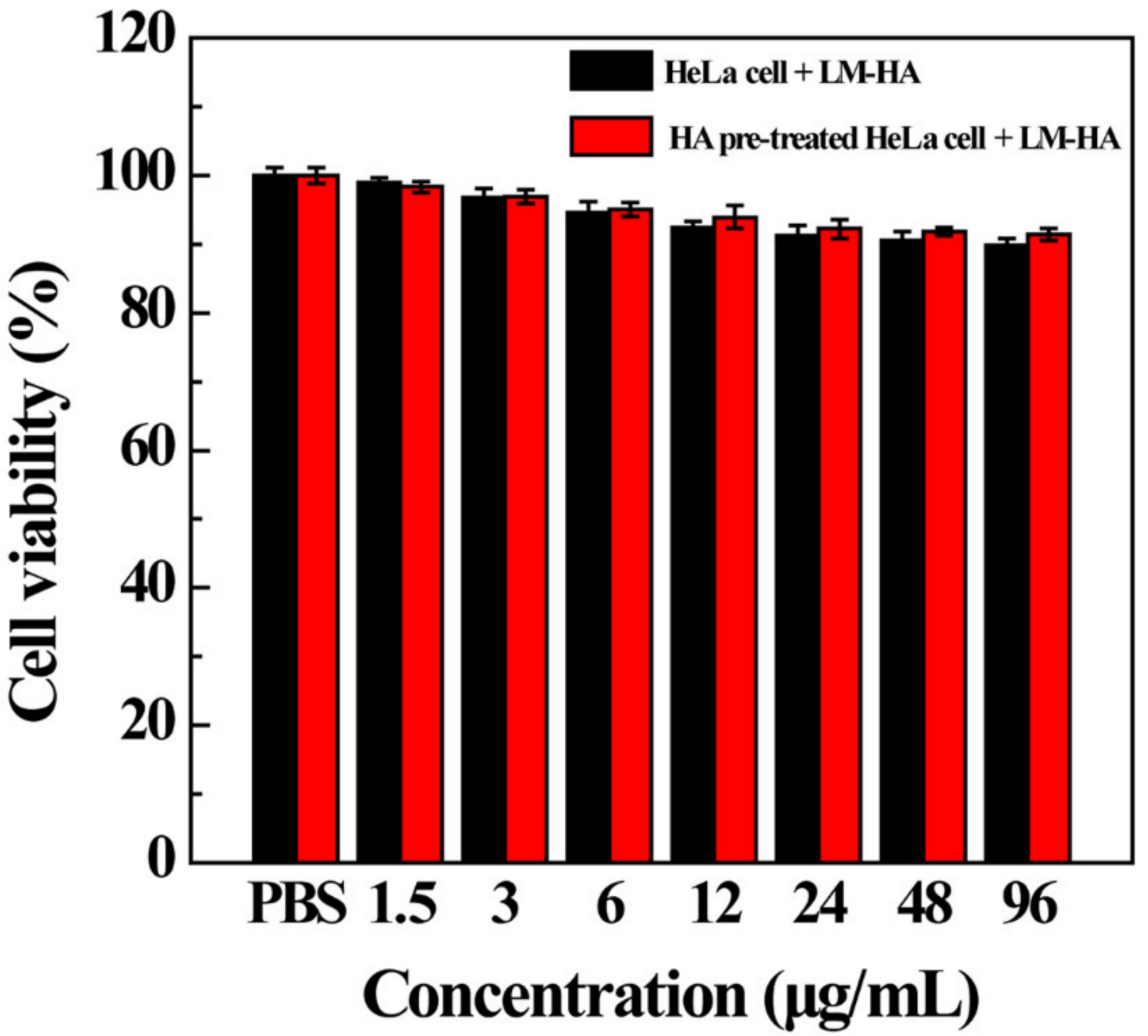
MTT viability assay of HeLa cells and HA pre-treated HeLa cells treated with LM-HA for 24 h, respectively.

To evaluate the targeted therapeutic efficacy of LM-HA/DOX, HeLa cells with overexpressing CD44 receptors were used as model cells, and free HA-pretreated HeLa cells were also prepared as a block control by culturing cells in HA-containing DMEM for 1 h before the experiment. Firstly, an MTT assay was conducted to evaluate the cytotoxicity of LM-HA on HeLa cells and HA-pretreated HeLa cells (Figure 6). It was obvious that the viability of both cells could remain over 90% after being treated with LM-HA within the concentration range of 1.5 to 96 μg/mL, demonstrating the good biocompatibility of the LM-HA nanocomposites.

**Figure 7:**
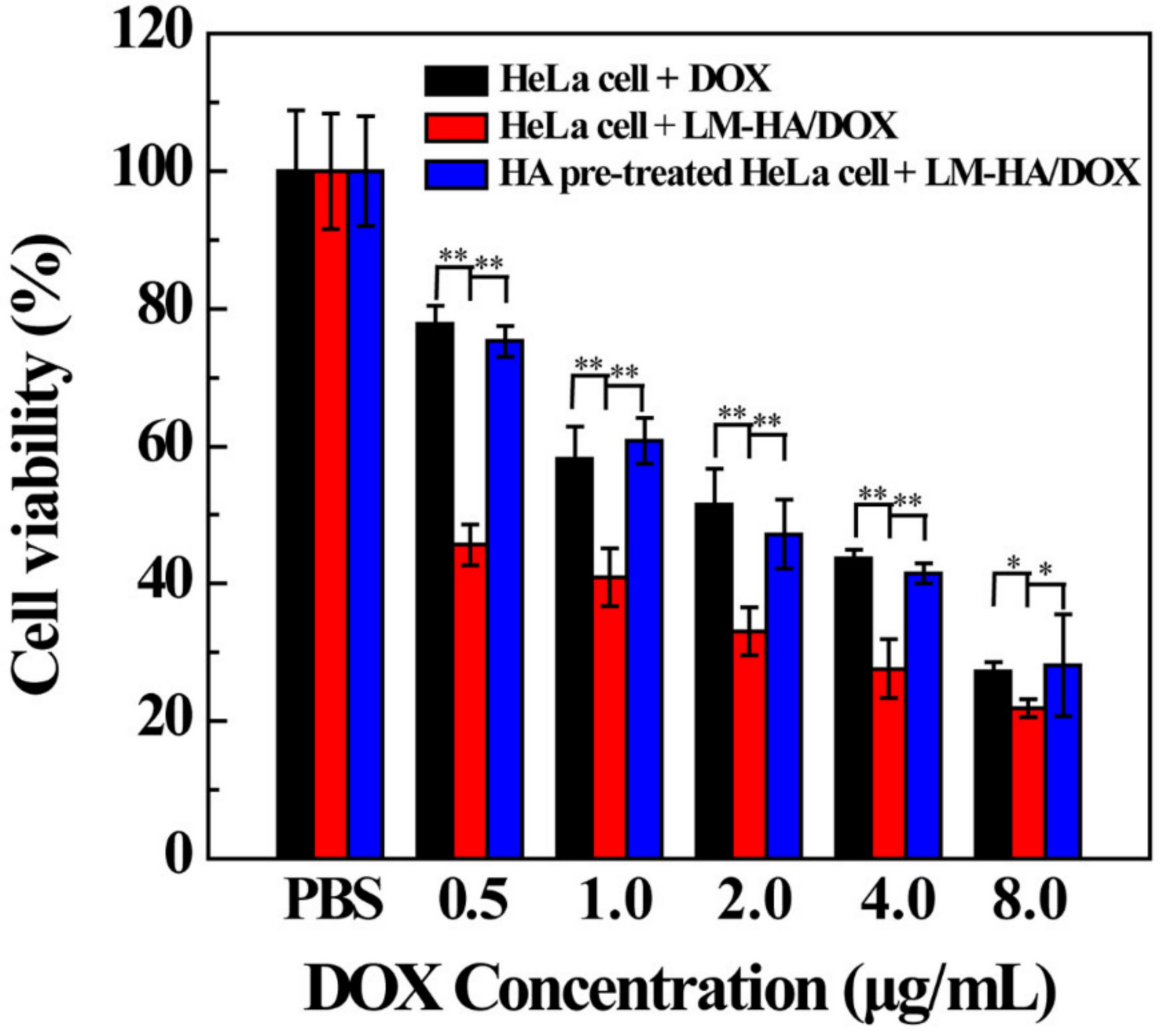
MTT viability assay of HeLa cells and HA pre-treated HeLa cells treated with free DOX and LM-HA/DOX nanocomposites for 24 h with different DOX concentrations.

To evaluate the therapeutic efficacy of LM-HA/DOX nanocomposites, the cell viability of HeLa cells was assessed by MTT assay after being treated with free DOX and LM-HA/DOX with different DOX concentrations for 24 h, and the viability of HA-pretreated HeLa cells treated with LM-HA/DOX was also measured (Figure 7). Both free DOX and LM-HA/DOX could restrain the proliferation of HeLa cells in a dose-dependent manner, demonstrating the prominent chemotherapeutic effect of DOX. Moreover, LM-HA/DOX displayed a significantly enhanced inhibition of HeLa cells in comparison with free DOX at the same DOX concentration, demonstrating the superior therapeutic effect of LM-HA/DOX. Importantly, the cell viability of HA-pretreated HeLa cells was much higher than that of HeLa cells when treating with the same dose of LM-HA/DOX. The half-maximal inhibitory concentration (IC_50_) of LM-HA/DOX in treating HA-pretreated HeLa cells (1.62 μg/mL) was found to be about 3.38 folds higher than that of HeLa cells (0.48 μg/mL), demonstrating that LM-HA/DOX could specifically inhibit HeLa cells via the CD44-mediated targeting. In summary, LM-HA/DOX nanocomposites can exert enhanced antitumor efficacy on cancer cells overexpressing CD44 receptors.

Finally, the targeted antitumor efficacy of the LM-HA/DOX nanocomposites was confirmed by morphology observation (Figure S2). Obviously, with the increase of DOX concentration, an increasing portion of HeLa cells and HA-pretreated HeLa cells became rounded and non-adherent, confirming the inhibition efficiency of LM-HA/DOX. In addition, LM-HA/DOX exhibited a higher inhibition rate in treating HeLa cells in comparison with HA-pretreated HeLa cells. This result is consistent with the MTT assay results, demonstrating the targeted inhibition of CD44-overexpressed cancer cells by LM-HA/DOX. 

### 3.4. CD44-Mediated Cellular Uptake

**Figure 8:**
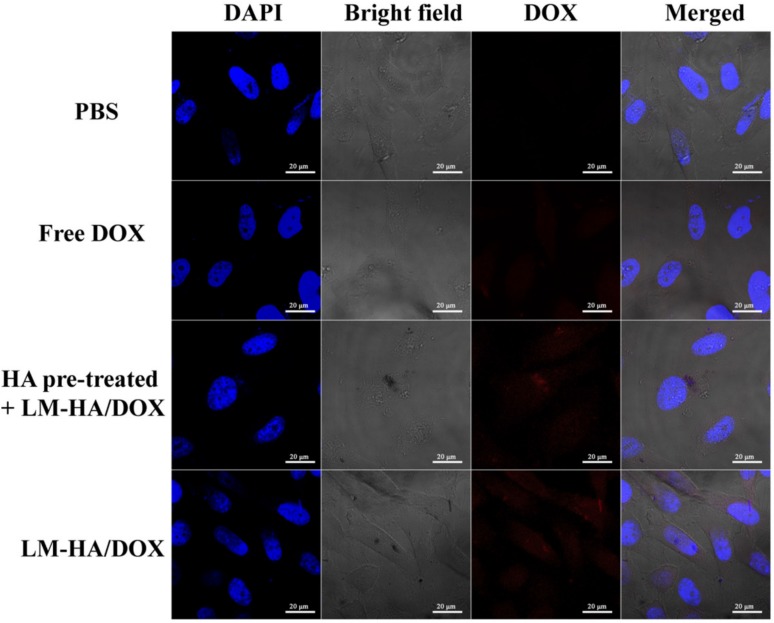
CLSM images of HeLa cells and HA pre-treated HeLa cells treated with PBS, free DOX and LM-HA/DOX nanocomposites for 4 h, at same DOX concentrations of 0.5 µg/mL.

HeLa cells and HA-pretreated HeLa cells were incubated with LM-HA/DOX nanocomposites for 4 h to assess the intracellular uptake of LM-HA/DOX by CLSM observation (Figure 8). The cell nuclei displayed blue fluorescence after being stained by DAPI, and the uptake and distribution of LM-HA/DOX could be illustrated by the self-fluorescence of DOX. Compared with the PBS control group, HeLa cells displayed DOX-related fluorescence in cell nuclei and cytoplasma after treated with free DOX and LM-HA/DOX. And the red fluorescence of LM-HA/DOX group was brighter than that of free DOX group, indicating the effective uptake of LM-HA/DOX by cells. Additionally, the red fluorescence signal accumulated in HA pre-treated HeLa cells was lower than that in HeLa cells, which was possibly due to the blockage of CD44 receptors on the cell surface by free HA. This outcome verified that the modification of HA could enhance the cellular uptake of LM-HA/DOX through CD44-mediated specific targeting.

**Figure 9:**
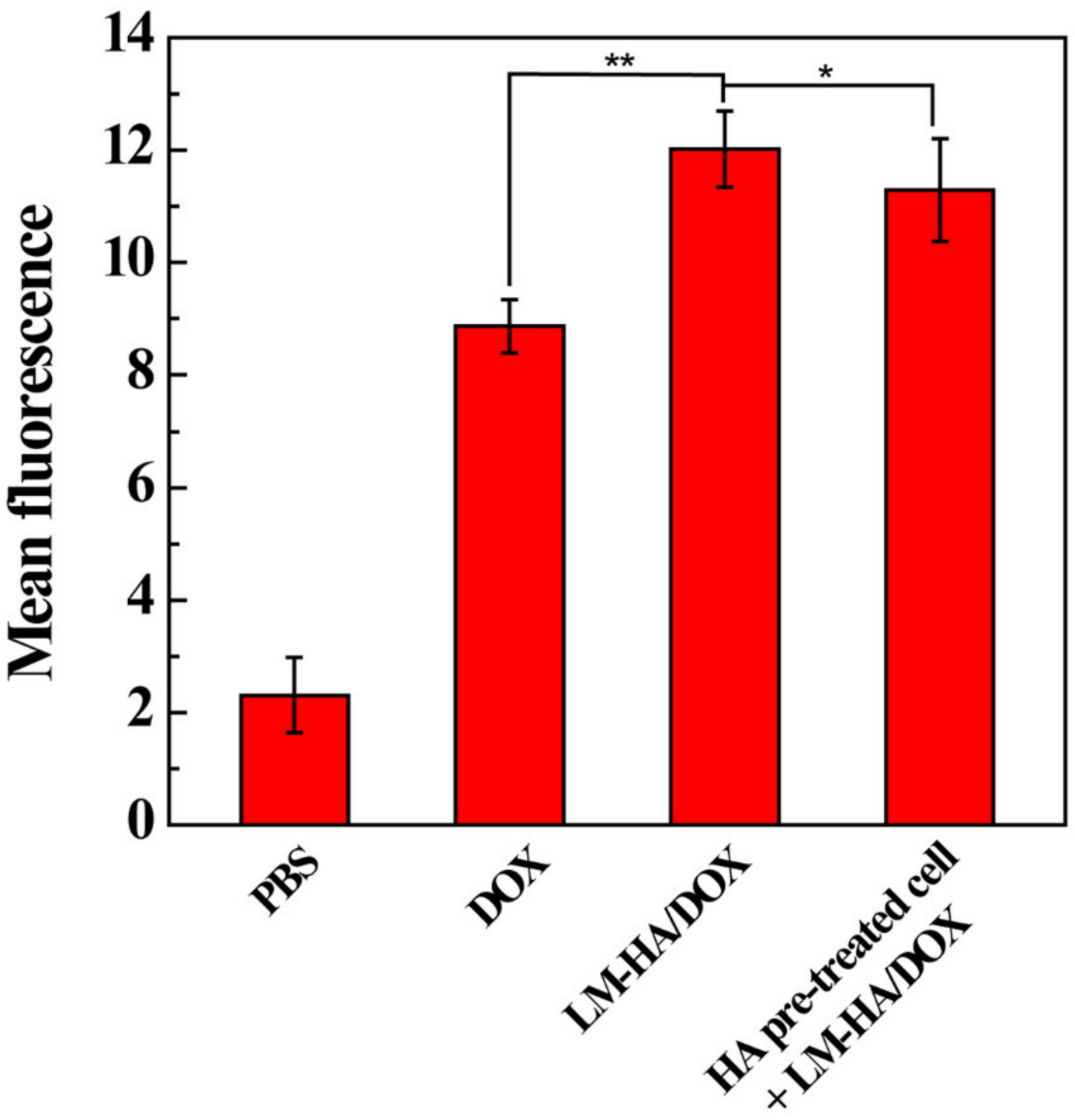
Mean fluorescence intensity of HeLa cells and HA pre-treated HeLa cells treated with PBS, free DOX and LM-HA/DOX nanocomposites for 4 h, at the same DOX concentrations of 0.5 µg/mL.

Finally, flow cytometry measurement was performed to further demonstrate the targeting specificity of LM-HA/DOX in HeLa cells (Figure 9). When compared to PBS control, both DOX and LM-HA/DOX displayed an obvious mean fluorescence enhancement, indicating the uptake of DOX and LM-HA/DOX by HeLa cells. Moreover, HeLa cells exhibited a significant enhanced mean fluorescence after being treated with LM-HA/DOX in comparison to HA pre-treated HeLa cells, in consistent with CLSM result. This phenomenon clearly demonstrated that HA modification could increase the cellular uptake by CD44-mediated endocytosis. Overall, LM-HA/DOX could selectively bind and internalize into HeLa cells due to the specific interaction between HA and CD44 overexpressed on cell surface, resulting in enhanced therapeutic efficacy in treating CD44-overexpressed cancer cells.

## 4. Conclusions

To conclude, hyaluronic acid-decorated LAP nanocomposites were successfully synthesized for targeted delivery of anticancer drugs to tumor cells overexpressing CD44 receptors. Obviously, the prepared LM-HA could load DOX effectively (85.1 ± 1.5%), and release drug in a pH-sensitive profile. In vitro cell viability assay, FCM, and CLSM results demonstrated that LM-HA/DOX nanocomposites exhibited a significantly enhanced antitumor efficacy than free DOX in treating CD44-overexpressed HeLa cells, due to their specific delivery and enhanced uptake via CD44-mediated endocytosis. Therefore, the designed LM-HA/DOX nanocomposites can be considered as a promising targeted anticancer drug delivery system for cancer treatment.

## Figures and Tables

**Table 1:** ζ-potential and hydrodynamic diameter of LAP, LM-NH_2_, LM-HA, and LM-HA/DOX, respectively. All the samples were dispersed in water at the concentration of 0.5 mg/mL.

Materials	ζ-Potential (mV)	Hydrodynamic Size (nm)
LAP	−39.6 ± 0.7	86.5 ± 5.1
LM-NH_2_	−12.0 ± 2.0	184.0 ± 1.9
LM-HA	−14.1 ± 1.4	282.6 ± 12.7
LM-HA/DOX	−10.8 ± 0.7	412.7 ± 16.1

## Supplementary Materials

The following are available online at http://www.mdpi.com/2073-4360/11/1/137/s1. Figure S1. Photographs of LM-HA/DOX dispersed in different solvents, Figure S2. Micrographs of HeLa cells and HA pre-treated HeLa cells treated with PBS, LM-HA/DOX for 24 h with different concentrations of DOX (1.0, 2.0, and 4.0 μg/mL), Table S1. ζ-potential and hydrodynamic diameter of LM-HA/DOX dispersed in water, PBS, DMEM, and FBS, respectively.
