# Supplementary material for: Hyaluronic Acid-Decorated Laponite® Nanocomposites for Targeted Anticancer Drug Delivery
Source: Polymers (Basel). 2019 Jan 14;11(1):137. doi: 10.3390/polym11010137 (PMC6401931; doi:10.3390/polym11010137)
Supplement: Supplementary file 1 [file polymers-11-00137-s001.pdf]

Supplementary Materials

Hyaluronic Acid-Decorated Laponite® Nanohybrids for Targeted Anticancer Drug Delivery

Tingting Jiang , Guangxiang Chen , Xiangyang Shi * and Rui Guo *

Key Laboratory of Science & Technology of Eco-Textile, Ministry of Education, College of Chemistry, Chemical Engineering and Biotechnology, Donghua University, Shanghai 201620, China; tingting_jiang1993@foxmail.com (T.J.); gxchen0429@163.com (G.C.)

* Correspondence: xshi@dhu.edu.cn (X.S.); ruiguo@dhu.edu.cn (R.G.); Tel.: +86-21-67792656 (X.S.); Tel.: +86-21-67792750 (R.G.)

Keywords: LAP; hyaluronic acid; doxorubicin; CD44 receptor targeted

1. Part of experimental section

1.1 Materials and Characterization Techniques

LAP with the formula $Na^{+0.7}[(Si_8Mg_{5.5}Li_{0.3})O_{20}(OH)_4]^{-0.7}$ was purchased from Zhejiang Institute of Geologic and Mineral Resources (Hangzhou, China). Doxorubicin hydrochloride (DOX.HCl) was purchased from Beijing Huafeng Pharmaceutical Co., Ltd. (Beijing, China), respectively. The 3-aminopropyldimethylethoxysilane (APMES), 1-ethyl-3-(3-dimethylaminopropyl) carbodiimide hydrochloride (EDC) and N-hydroxysuccinimide (NHS) were from J&K Chemical Ltd. (Shanghai, China). Hyaluronic acid (HA) was provided by Sinopharm Chemical Reagent Co., Ltd (Shanghai, China). HeLa cells (a human cervical cancer cell line) were obtained from the Institute of Biochemistry and Cell Biology (Chinese Academy of Sciences, Shanghai, China).

ζ-potential and dynamic light scattering (DLS) measurements were performed using a ZetasizerNano ZS system (ZEN3600, Malvern, Worcestershire, UK) equipped with a standard He-Ne laser (λ = 633 nm) to analyze the fundamental properties of the samples. To evaluate the colloidal stability of different samples, the hydrodynamic sizes of the samples were also measured after they were dispersed in different solvents. UV-vis spectroscopy was carried out using a Lambda 25 UV-vis spectrophotometer (PerkinElmer, Waltham, MA). Thermal gravimetric analyses (TGA) of different samples were performed on a TG 209 F1 thermogravimetric analyzer (NETZSCH, Selb/Bavaria, Germany) from room temperature to 900 ^{o}C at a heating rate of 10 ^{o}C/min under nitrogen atmosphere. Fourier transform infrared (FT-IR) spectra were collected on a Nexus 670 spectrometer (Thermo Nicolet Corporation, Madison, WI).

1.2 In Vitro Drug Release

The in vitro release kinetics of DOX from LM-HA/DOX nanocomposites under different pH conditions were monitored using UV-vis spectrometry. Briefly, LM-HA/DOX nanocomposites (1 mg) dispersed into 1 mL PBS solution (pH = 7.4) or 1 mL citric acid-disodium hydrogen phosphate buffer (pH = 5.0) were placed in a dialysis bag with MWCO of 8000-14000, and suspended in the corresponding buffer medium (9 mL). All samples were incubated in a vapor-bathing constant temperature vibrator at 37 ^{o}C. At the predetermined time interval, 1 mL buffer solution was taken out and an equal volume of the corresponding buffer solution was replenished. The released DOX was quantified using UV-vis spectroscopy at 480 nm.

1.3 Statistical Analysis

One way analysis of variance (ANOVA) statistical analysis was used to assess the significance of the experimental data by selecting a p value of 0.05 as a significance level. The data were indicated with $*p < 0.05$, $**p < 0.01$, and $***p < 0.001$, respectively.

2. Results

Table S1. ζ-potential and hydrodynamic diameter of LM-HA/DOX dispersed in water, PBS, DMEM, and FBS, respectively.

Materials	ζ-potential (mV)	Hydrodynamic size(nm)	Polydispersity index (PDI)
LM-HA/DOX (water)	-10.8 ± 0.7	412.7± 16.1	0.312 ± 0.095
LM-HA/DOX (PBS)	-9.3 ± 0.5	445.7 ± 10.1	0.401 ± 0.041
LM-HA/DOX (DMEM)	-10.2 ± 1.8	480.3± 9.7	0.358 ± 0.039
LM-HA/DOX (FBS)	-11.8 ± 1.9	469.4± 11.2	0.394 ± 0.074

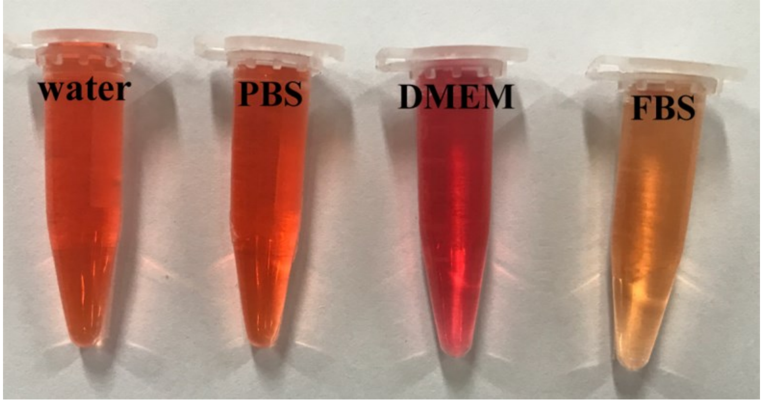

Figure S1. Photographs of LM-HA/DOX dispersed in different solvents.

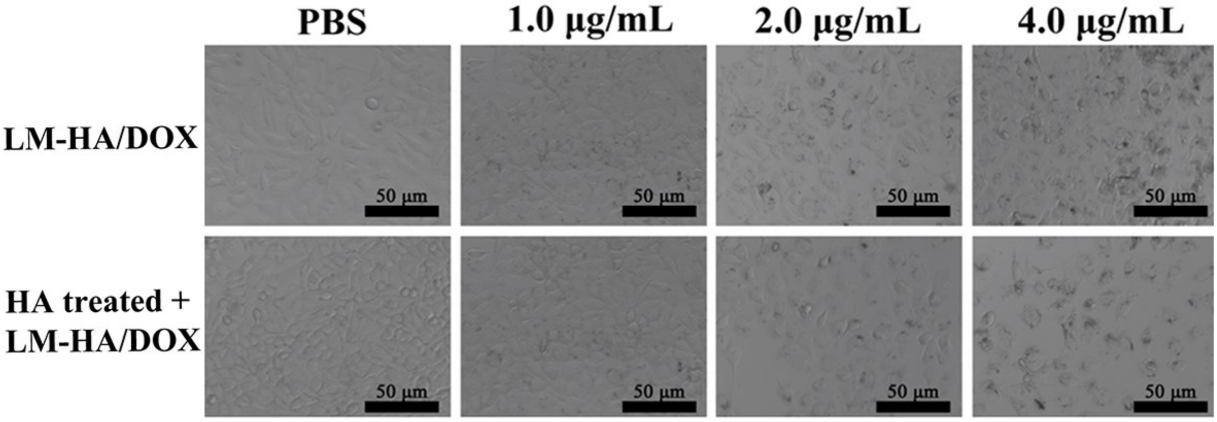

Figure S2. Micrographs of HeLa cells and HA pre-treated HeLa cells treated with PBS, LM-HA/DOX for 24 h with different concentrations of DOX (1.0, 2.0, and 4.0 μg/mL).
